# Animal species identification in parchments by light

**DOI:** 10.1038/s41598-019-38492-z

**Published:** 2019-02-12

**Authors:** Angel Martin Fernandez Alvarez, Julie Bouhy, Marc Dieu, Catherine Charles, Olivier Deparis

**Affiliations:** 1 Heritages, Transmissions, Inheritances (PaTHs) Institute & Department of Physics, University of Namur, Namur, Belgium; 2 Mass Spectrometry Facility (MaSUN), University of Namur, Namur, Belgium; 3 Moretus Plantin University Library, University of Namur, Namur, Belgium

## Abstract

Recently, historical and conservation studies have attached an increasing importance to investigating the materials used in historic documents. In particular, the identification of the animal species from which parchments are made is of high importance and is currently performed by either genetic or proteomic methods. Here, we introduce an innovative, non-invasive optical method for identifying animal species based on light-parchment interaction. The method relies on conservation of light energy through reflection, transmission and absorption from the sample, as well as on statistical processing of the collected optical data. Measurements are performed from ultraviolet (UV) to near-infrared (NIR) spectral ranges by a standard spectrophotometer and data are processed by Principal Component Analysis (PCA). PCA data from modern parchments, made of sheep, calf and goat skins, are used as a database for PCA analysis of historical parchments. Using only the first two principal components (PCs), the method confirmed visual diagnostics about parchment appearance and aging, and was able to recognise the origin species of historical parchment of among database clusters. Furthermore, taking into account the whole set of PCs, species identification was achieved, with all results matching perfectly their proteomic counterparts used for method assessment. The validated method compares favourably with genetic and proteomic methods used for the same purpose. In addition to animals’ proteomic and genetic signatures, a unique “optical fingerprint” of the parchments’ origin species is revealed here. This new method is non-invasive, straightforward to implement, potentially cheap and accessible to scholars and conservators, with minimal training. In the context of cultural heritage, the method could help solving questions related to parchment production and, more generally, medieval writing production.

## Introduction

**Figure 1:**
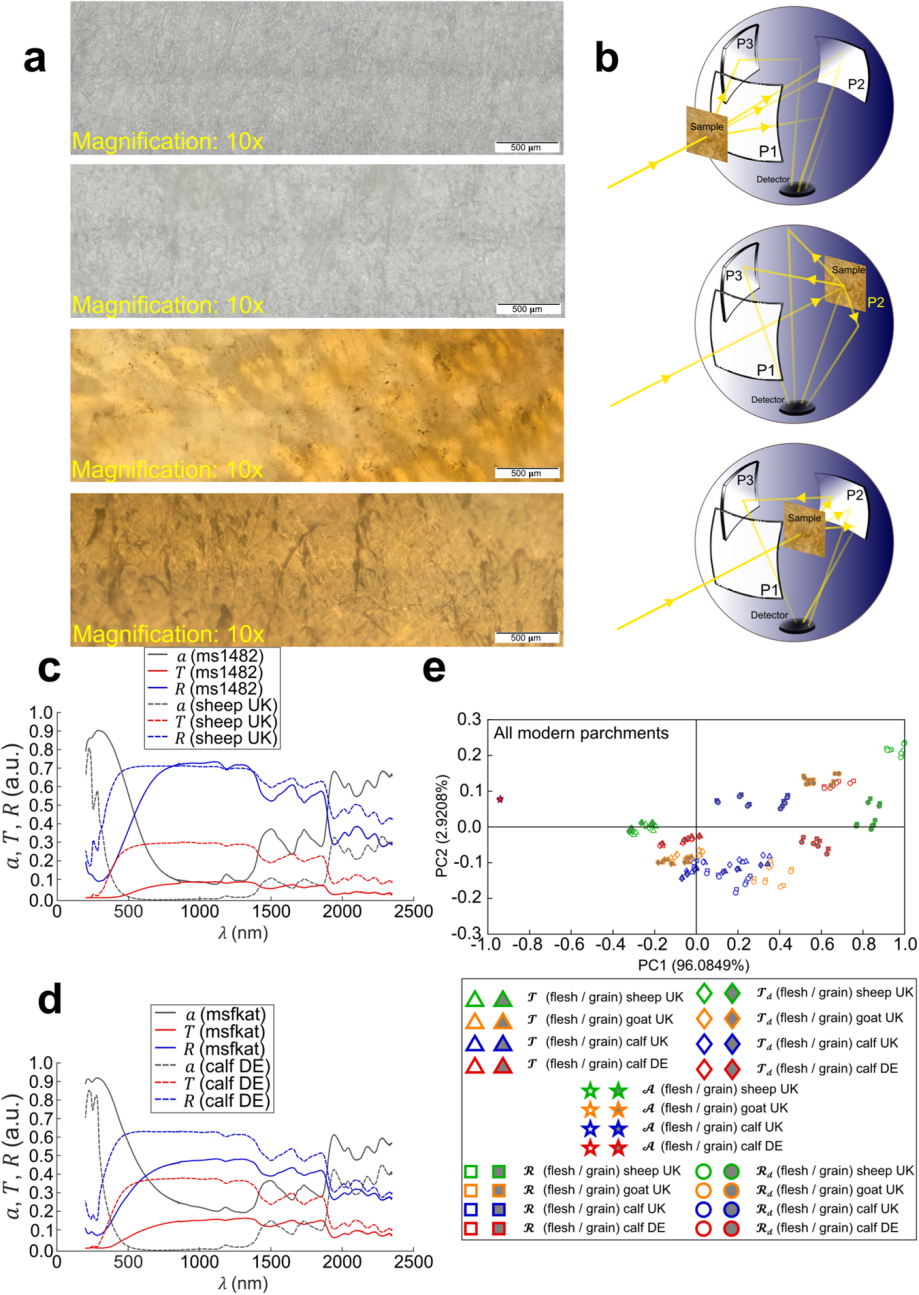
Determination of animal origin species in parchments by spectrophotometry combined with PCA data processing. (**a**) Stitched microscope images (10× magnification) of (from top to bottom) a modern sheep parchment (flesh side, grain side) and an historical (ms1482) parchment (recto, verso). (**b**) Schematic of experimental set-up configurations for total transmittance (top), total reflectance (middle) and absorbance measurements (bottom). An integrating sphere is used as detector in a standard double-beam spectrophotometer (abbreviations; P1, P2, P3 for port 1, 2 and 3, respectively. See details in Methods). (**c**,**d**) Total transmittance, total reflectance and absorption spectra measured (arbitrary units: a.u.) on modern sheep (**c**) and calf (**d**) parchment samples as well as on historical samples of the same species (determined independently by a proteomic method). (**e**) PCA representation for all observations of all modern parchment samples (grain side and flesh side), used as species database for identification. The percentage variability explained (PVE) is displayed for PC1 and PC2 on each axis.

The knowledge of the animal origin of parchment folios is a question of great interest in codicology: a discipline that, traditionally, studies the materiality of manuscript books, i.e. codices^1^. In a broader context, the identification of the origin species of parchments allows historians to confirm theories about medieval manuscript production, as well as parchment production and trade. Accurate species identification can help scholars considering long-standing controversies on, for instance, the origin of uterine vellum used in 13th century codices^2–5^. In view of cultural heritage preservation, determination of the origin species of parchment, by means of other than visual inspection of e.g. faded traces of hair follicles patterns, could also help conservators adapting storage conditions of historical parchments. Parchment is made from the dermis of animal skins, treated in such a way that chemical processes responsible for the decomposition of organic compounds stop^6^. The most frequently used animals are goat, calf and sheep^7^. At macroscopic scale, both sides of the parchment are distinguishable (Fig. 1a), at least, in good quality parchments^8,9^; on the grain side, hair follicles are visible, whereas, on the flesh side, the surface looks more homogeneous. At a microscopic scale, parchment is mainly composed of type I collagen fibres oriented parallel to the surface and organised in a network architecture (Fig. 1a). Each fibre is made of fibrils, themselves composed by several thousands of triple helices of collagen proteins, consisting of species-specific sequences of amino acids^10,11^. Hair follicles patterns and skin fat content, which influence, respectively, the appearance and texture of the surface, are characteristic of the animal species^12,13^. However, successive treatments of the animal skin during parchment manufacture and surface degradation over time make determining the species through visual inspection and palpation difficult. Therefore, developing reliable methods to identify species correctly is very desirable.

The non-invasive “ZooMS” proteomic method^2^ has been shown to be successful in identifying animal species that are the most relevant for parchment studies^3^. Recently, a non-destructive DNA method was developed based on the same sampling protocol^2^, providing additional information, such as the animal gender^4^. Invasive DNA methods have also been used^14,15^. These techniques are reliable but expensive and time consuming, due to sample preparation^16^. On the other hand, optical methods are intrinsically non-invasive and require no preparation. They are already employed to evaluate the stage of degradation of parchments^17,18^ or to distinguish between modern, historical and artificially aged parchments^19^. The use of optics is possible because the collagen and its structural organisation provide parchment with birefringence, non-linear optical properties and fluorescence^17,19,20^. Due to its structure and composition, parchment scatters and absorbs light, so that diffuse reflection and transmission (as well as absorption) can be observed. However, no optical method has been proposed so far to determine the animal origin of parchments. Here, we introduce a novel method for the identification of a parchment’s origin species, which combines optics and statistics. Basically, absorption and scattering properties are measured over a broad spectral range (UV-visible-NIR), using standard photospectrometry equipment (Fig. 1b). The collected optical data (Fig. 1c,d) are treated using principal component analysis (PCA) (Fig. 1e), a well-known statistical method previously used for characterization of parchment degradation^21–23^. The proposed method can lead to accurate species identification, even for closely related species (goat versus sheep), whose collagen proteins differ by only a few amino acids. It is noteworthy that, recently, a method based on invasive time-of-flight secondary ion mass spectrometry measurements combined with PCA showed interesting potentiality for species recognition in parchments^24^. By contrast, our non-invasive method can be made portable by using a fibre optic photospectrometer and standard optical accessories. Furthermore, it can be easily used by trained people in libraries and museums. Those advantages are very attractive in cultural heritage science, where fragile and precious manuscripts need to be examined under stringent conditions, in order to preserve them.

## Results

In order to show the consistency of the method, we measured the following on a set of modern parchments (21 samples) and historical parchments (20 samples) – diffuse reflectance and transmittance (*R*_*d*_, *T*_*d*_), hemispherical (total) reflectance and transmittance (*R*, *T*) and absorbance (*A*) over a broad wavelength (*λ*) range, from UV (200 nm) to NIR (2350 nm), using a standard spectrophotometer equipped with an integrating sphere (Fig. 1b). It is noteworthy that sample absorption (*a*) is related to measured absorbance (see details in Methods). As far as light-matter interaction is concerned, total reflectance, total transmittance and absorption are entangled spectral quantities, i.e. they obey the energy conservation law: $a(\lambda )+R(\lambda )+T(\lambda )=1,\,\forall \lambda$. The method uses PCA in order to eliminate redundancy and noise from measurements^25^. In order to distinguish measured quantities from their PCA representation, we use, respectively, capital Roman letters (*A*, *T*, *T*_*d*_, *R*, *R*_*d*_) and script letters ($\pmb{\mathscr{A}}$, $\pmb{\mathscr{T}}$, ${\pmb{\mathscr{T}}}_{{\boldsymbol{d}}}$, ${\boldsymbol{ {\mathcal R} }}$, ${{\boldsymbol{ {\mathcal R} }}}_{{\boldsymbol{d}}}$).

### Recovery of underlying light-matter interaction

**Figure 2:**
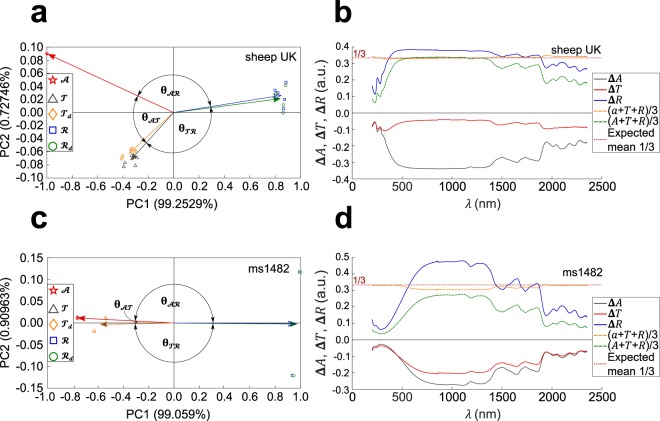
PCA data representation from *A*, *T*, *T*_*d*_, *R*, *R*_*d*_ measurements on modern and historical parchment samples. (**a**,**c**) PCA representation of modern sheep (Cowley, UK) parchment data (**a**) and historical (ms1482) parchment data (**c**). For each type of measured quantity, a centroid vector is calculated from data acquired on several samples. The angle **θ** between centroid vectors is related to Pearson coefficient by r = *cos*θ. For each PCA representation, the percentage variability explained (PVE) is displayed on each axis. (**b**,**d**) Spectral variations (arbitrary units: a.u.) of absorbance (Δ*A*), total reflectance (Δ*R*), total transmittance (Δ*T*) measured on, respectively, flesh side and recto, of the same modern (**b**) and historical (**d**) samples. The horizontal line is the mean value between the three quantities expected from energy conservation, i.e. 1/3.

In order to prove that this method is able to recover the underlying physics of light-parchment interaction, spectral data collected from all modern and historical parchments were organized in a data matrix *X* of size *M* × *N*, where *M* is the number of observations (measurements) and *N* is the number of variables (wavelengths). It is crucial to note that, in what follows, the number of measurements that is taken into account in *X* is varied on purpose, according to the goals of our analyses. Specifically, a matrix *X* is built for every parchment sample under test, which is composed of submatrices *X*_*α*_ of size *p* × *N*, where *α* identifies the total number of samples (*n*) (See details in Methods). Each submatrix *X*_*α*_ contains the five types of spectral measurements (*p* = 5). For instance, a modern sheep parchment from Cowley, UK (*n* = 5 samples) is associated with a matrix *X* of *M* = *n* × *p* = 25 observations (measurements) and *N* = 2150 variables (wavelengths). PCA representation of the data is shown for a modern parchment and a historical parchment (Fig. 2a,c). Sample data clusters ($\pmb{\mathscr{A}}$, $\pmb{\mathscr{T}}$, ${\pmb{\mathscr{T}}}_{{\boldsymbol{d}}}$, ${\boldsymbol{ {\mathcal R} }}$, ${{\boldsymbol{ {\mathcal R} }}}_{{\boldsymbol{d}}}$) associated with each type of measurements are characterized by a centroid vector and Pearson’s coefficient (−1 ≤ *r* ≤ 1) expresses the degree of correlation between those. Alternatively, any angle **θ** (${{\boldsymbol{\theta }}}_{\pmb{\mathscr{A}}\pmb{\mathscr{T}}}$, ${{\boldsymbol{\theta }}}_{\pmb{\mathscr{T}}{\boldsymbol{ {\mathcal R} }}}$, ${{\boldsymbol{\theta }}}_{\pmb{\mathscr{A}}{\boldsymbol{ {\mathcal R} }}}$) between vectors can be used since *r* = cos θ. Parallel vectors (θ = 0) and anti-parallel vectors (θ = π) correspond to positively correlated (*r* = 1) and negatively correlated (*r* = −1) quantities, respectively. Since PC1 and PC2 form an orthonormal basis set^25^, vectors aligned along PC1 and PC2 axes (θ = π/2) correspond to no correlation.

In both modern and historical sheep parchments, PCA representations of *R*, *T* and *R*_*d*_, *T*_*d*_ data are positively correlated, i.e. the corresponding centroid vectors are close together (Fig. 2a,c). This is expected from the fact that parchment surface is essentially diffusive, i.e. it scatters light in all directions, so that specular contribution to reflection is small, hence *R*_*d*_ ≅ *R* (and therefore ${{\boldsymbol{ {\mathcal R} }}}_{{\boldsymbol{d}}}$ ≅ ${\boldsymbol{ {\mathcal R} }}$) and *T*_*d*_ ≅ *T* (and therefore ${\pmb{\mathscr{T}}}_{{\boldsymbol{d}}}$ ≅ $\pmb{\mathscr{T}}$) Moreover, PCA representations of *R*, *R*_*d*_, *T*, *T*_*d*_ and *A* are negatively correlated to each other (Fig. 2a,c). These results, which were also found for modern and historical calf parchments (Supplementary Figures S1–S7), are consistent with energy conservation and with the underlying physics of light-parchment interaction in the presence of scattering and absorption. Indeed, looking at spectral variations of the measured quantities (Fig. 2b,d), it turns out that, for both modern and historical parchments, variations of *R* and *A* are of opposite signs over the entire spectrum. The same trend holds between variations of *R* and *T*. On the other hand, variations of *A* and *T* are of identical signs over the entire spectrum. Those observations are fully consistent with the relative positions of vectors (**θ** angles) in PCA representation (Fig. 2a,c), which show that ${\boldsymbol{ {\mathcal R} }}$ and $\pmb{\mathscr{A}}$ as well as ${\boldsymbol{ {\mathcal R} }}$ and $\pmb{\mathscr{T}}$ tend to be negatively correlated (Pearson coefficients for ms1482: *r* = −0.9999 and *r* = −1.0000, respectively. See values for other samples in Table 1) whereas $\pmb{\mathscr{A}}$ and $\pmb{\mathscr{T}}$ tend to be positively correlated (*r* = 0.9998). Moreover, energy conservation imposes a constraint on the problem: variations in *A*, *R* and *T* must balance each other. The quantity, $\frac{1}{3}[A(\lambda )+R(\lambda )+T(\lambda )]$, however, is not exactly the expected one (1/3), simply because absorbance is measured, rather than absorption. If *A*(*λ*) values are converted into *a*(*λ*) values, an almost perfect balance is achieved over the whole spectrum, i.e. $\frac{1}{3}[a(\lambda )+R(\lambda )+T(\lambda )]\cong \frac{1}{3},\,\forall \lambda$ (Fig. 2b,d). It is noteworthy that this balance holds for any number of arbitrary samples included in the *X* matrix. Imperfect balance tends to exist in spectral regions where parchment absorption is strong (below 500 nm and above 1300 nm), whereas both absorption and absorbance are almost the same in the 500–1300 nm range where parchment is more transparent (small absorption).

**Table 1:** Pearson’s correlation coefficients.

Parchments (ms)*	${{\boldsymbol{\theta }}}_{\pmb{\mathscr{A}}{{\bf{T}}}^{\ast \ast }}$	${{\boldsymbol{r}}}_{\pmb{\mathscr{A}}{\bf{T}}}$	${{\boldsymbol{\theta }}}_{\pmb{\mathscr{A}}{{\boldsymbol{ {\mathcal R} }}}^{\ast \ast }}$	${{\boldsymbol{r}}}_{\pmb{\mathscr{A}}{\boldsymbol{ {\mathcal R} }}}$	${{\boldsymbol{\theta }}}_{\pmb{\mathscr{T}}{{\boldsymbol{ {\mathcal R} }}}^{\ast \ast }}$	${{\boldsymbol{r}}}_{\pmb{\mathscr{T}}{\boldsymbol{ {\mathcal R} }}}$
sheep UK flesh side	17.62	0.9531	172.99	−0.9925	169.39	−0.9829
sheep UK grain side	9.00	0.9877	176.17	−0.9978	174.83	−0.9959
calf UK flesh side	161.21	−0.9467	162.83	−0.9555	35.96	0.8095
calf UK grain side	138.26	−0.7462	159.71	−0.9379	62.03	0.4689
goat UK flesh side	112.82	−0.3878	172.90	−0.9923	74.28	0.2709
goat UK grain side	57.12	0.5429	166.47	−0.9723	136.41	−0.7243
calf DE flesh side	39.06	0.7765	170.99	−0.9877	149.95	−0.8656
calf DE grain side	31.41	0.8534	174.97	−0.9961	153.62	−0.8959
mshap #°1	1.26	0.9998	179.34	−0.9999	179.40	−0.9999
mshap #°2	5.62	0.9952	176.58	−0.9982	177.80	−0.9993
ms1593 #°1	2.68	0.9989	178.30	−0.9996	179.02	−0.9999
ms1593 #°2	5.25	0.9958	176.90	−0.9985	177.85	−0.9993
ms1483 #°1	2.11	0.9993	178.75	−0.9998	179.14	−0.9999
ms1483 #°2	6.42	0.9937	176.67	−0.9983	176.91	−0.9985
msmeh #°1	6.31	0.9939	176.75	−0.9984	176.94	−0.9986
msmeh #°2	7.88	0.9906	175.87	−0.9974	176.25	−0.9979
msfkat #°1	8.90	0.9880	175.83	−0.9974	175.27	−0.9966
msfkat #°2	8.78	0.9883	175.59	−0.9970	175.63	−0.9972
ms1240 #°1	9.77	0.9855	175.46	−0.9969	174.77	−0.9958
ms1240 #°2	23.88	0.9144	171.68	−0.9895	164.44	−0.9634
ms1556 #°1	5.44	0.9955	176.84	−0.9985	177.72	−0.9992
ms1556 #°2	5.51	0.9954	176.93	−0.9986	177.56	−0.9991
ms1482 #°1	1.27	0.9998	179.21	−0.9999	179.52	−1.0000
ms1482 #°2	2.73	0.9989	178.23	−0.9995	179.04	−0.9999
ms499 #°1	0.70	0.9999	179.55	−1.0000	179.75	−1.0000
ms499 #°2	3.81	0.9978	177.75	−0.9992	178.44	−0.9996
msopph #°1	3.79	0.9978	177.56	−0.9991	178.65	−0.9997
msopph #°2	3.93	0.9977	177.69	−0.9992	178.38	−0.9996

*Ms is the abbreviation for manuscript.

**Angles are measured in degrees.

### Confirmation of visual diagnostics on parchments

Scholars and conservators have long developed ways to characterize parchment material status from visual inspection. We checked our method is consistent with their diagnostics as far as parchment appearance and aging are concerned.

**Figure 3:**
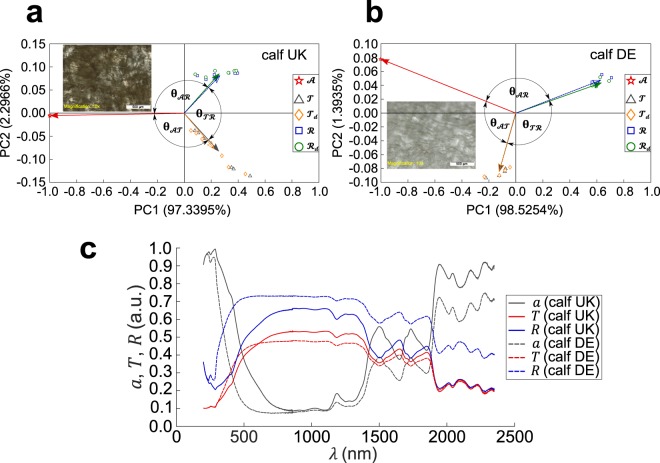
PCA data representation from *A*, *T*, *T*_*d*_, *R*, *R*_*d*_ measurements on modern calf parchment samples having light and dark appearance. (**a**,**b**) PCA representation of dark parchment (Cowley, UK) data (**a**) and light parchment (Schmedt, DE) data (**b**). Inset: microscope images of samples. The percentage variability explained (PVE) is displayed for PC1 and PC2 on each axis. (**c**) Absorption, total reflectance, total transmittance (arbitrary units: a.u.) measured on flesh sides of the same samples as in (**a**,**b**).

Parchment appearance may vary from dark to light according to the animal species, the manufacture process and natural aging. For instance, modern calf parchment from Cowley, UK (Fig. 3a) appears dark, whereas modern calf parchment from Schmedt, DE appears light (Fig. 3b). PCA representations of *R* and *T* (Fig. 3a,b) shows higher Pearson coefficient for dark parchment (*r* = 0.8095) than for light parchment (*r* = −0.8656), i.e. the angle between *R* and *T* vector is smaller for the former than the latter (${{\boldsymbol{\theta }}}_{\pmb{\mathscr{T}}{\boldsymbol{ {\mathcal R} }}}$ = 35.96° versus ${{\boldsymbol{\theta }}}_{\pmb{\mathscr{T}}{\boldsymbol{ {\mathcal R} }}}$ = 149.95°). In terms of light scattering, in the dark parchment, strong absorption of light imposes both low reflectance and transmittance (Fig. 3c), as required by energy conservation, which produces $\pmb{\mathscr{A}}$ vector opposed to both ${\boldsymbol{ {\mathcal R} }}$ and $\pmb{\mathscr{T}}$ vectors in PCA representation (Fig. 3a). In the light parchment, high reflectance and low absorption are responsible for low transmittance (Fig. 3c), thus explaining the relative positions of the corresponding vectors, i.e. ${\boldsymbol{ {\mathcal R} }}$ vector opposed to both $\pmb{\mathscr{A}}$ and $\pmb{\mathscr{T}}$ vectors (Fig. 3b). Those results are consistent with visual diagnostics about parchment appearance. Noticeably, the method allows us to distinguish light and dark parchments, confirming the basic visual inspection.

All historical parchments under test were found to exhibit a strong positive correlation between PCA representation of *A* and *T*, whereas, in all modern parchments, a significantly lesser degree of correlation (Pearson’s coefficient) was found (Table 1). The origin of strong positive correlation in historical parchments can be traced back to spectral variations of *A* and *T*, which closely follow each other over the whole spectrum (Fig. 2d). This difference in behaviour between modern and historical parchments does not depend on species. Rather, it is likely to be related to parchment’s natural aging. Aging is known to cause parchment gelatinization^20,26,27^, which leads to spreading of the UV absorption band into the visible range (Fig. 1c,d), hence changing *a*(*λ*), *R*(*λ*) and *T*(*λ*) spectra. Therefore, differences appear between optical spectra of modern and historical parchments: *a*(*λ*) increases in historical samples compared with modern samples; by contrast, *R*(*λ*) is roughly the same over the whole spectrum (Fig. 1c,d). Thus, by virtue of light energy conservation, transmittance decreases compared to pristine (modern) sample. Interestingly, the distinction between modern and historical parchments, which may be inferred from visual inspection by a trained person, is also possible thanks to our method.

### Recognition and identification of parchment origin species

Using a two-dimensional PCA representation, we first discovered that recognition of parchment’s origin species could be achieved using solely PC1 and PC2, thus unveiling the dynamics hidden in entangled optical measurements.

For each historical parchment, a *X* matrix was built. The first rows contained *A*, *T*, *T*_*d*_, *R*, data of all modern parchments, grain and flesh sides included, and the last rows contained data from the historical parchment under test. In practice, the *X* matrix consisted of *N* = 2150 variables and *M* = 5 × 46 = 230 observations, i.e. *p* = 5 spectral measurements and *n* = 46 samples (2 × 21 modern flesh side and grain side samples, 2 historical samples taken from the same folio on both sides).

**Figure 4:**
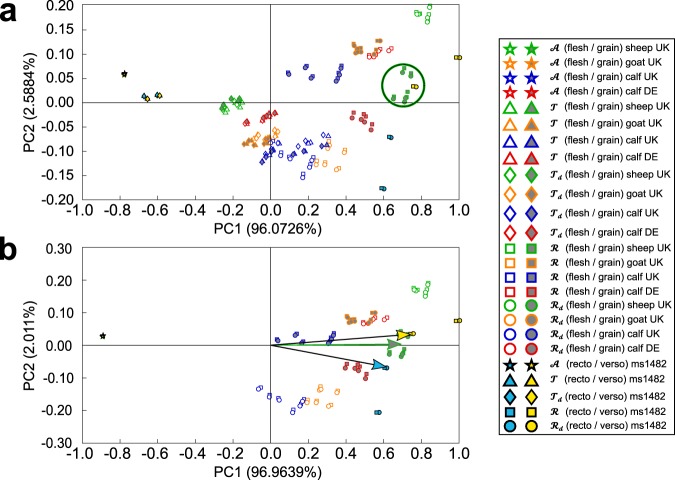
PCA data representation from measurements on all modern parchment samples and one historical sample. (**a**) All types of measurements (*A*, *T*, *T*_*d*_, *R*, *R*_*d*_) included in data set. The green circle delimits the recognition cluster. (**b**) Only 3 types measurements included in data set (*T*, *T*_*d*_ excluded). Species of the historical (ms1482) sample was determined independently by a proteomic method. Centroid vectors of recognition cluster and historical parchment (ms1482), at both sides (recto and verso) are displayed with respective colours. The percentage variability explained (PVE) is displayed for PC1 and PC2 on each axis.

In PC1-versus-PC2 two-dimensional representation, clusters are observed in modern parchment data for different species and skin sides (Fig. 4a). Clusters for flesh and grain sides are well separated for the most representative PC1 values, i.e. those associated with *R* and *R*_*d*_. On the other hand, data close to the centre of the plot are weakly representative for the first PC components. Therefore, *T* and *T*_*d*_ measurements do not bring significant contribution to PCA and can be discarded from matrix entries without affecting the recognition process. To confirm this, a new data matrix was built from 3 spectral measurements (*A*, *R*, *R*_*d*_) for the same samples (*M* = 184 and *N* = 2150) and PCA was recalculated (Fig. 4b). Data of the historical parchment under test are located around clusters of different species, according to the side of the sample (green circle in Fig. 4a), which hampers possible recognition of the species. In order to circumvent this problem, recognition is performed from the most representative data in PC1-PC2 representation, i.e. those which have the highest scores (see details in Methods). In order to quantify confidence in the species-recognition performed, the norm *d* of the difference between the historical sample vector and the modern sample cluster centroid vector (Fig. 4b) is calculated. A proximity percentage is defined as: $100 \% \times (1-d)$. For instance, the species of origin of historical (ms1482) parchment was found to be close to Cowley (UK) sheep parchment in our species database (Table 2, 3^rd^ column).

**Table 2:** PCA results.

Historical Parchment (ms)*	Proteomic Results	PCA Recognition (%proximity/side)	PCA Identification All samples** (%proximity/side)	PCA Identification Two samples** (%proximity/side)
mshap #°1	sheep	sheep UK (94.5%/flesh)	sheep UK (85.4%/flesh & 84.8%/grain)	sheep UK (85.4%/flesh & 84.7%/grain)
mshap #°2	sheep	sheep UK (94.5%/flesh)	sheep UK (87.1%/flesh & 84.1%/grain)	sheep UK (87.3%/flesh & 84.1%/grain)
ms1593 #°1	sheep or goat	calf UK (90.3%/grain)	sheep UK (79.6%/flesh & 79.9%/grain)	sheep UK (79.6%/flesh & 79.9%/grain)
ms1593 #°2	sheep or goat	calf UK (90.3%/grain)	sheep UK (83.5%/flesh & 83.3%/grain)	sheep UK (83.6%/flesh & 83.3%/grain)
ms1483 #°1	sheep	calf DE (96.2%/flesh)	sheep UK (86.9%/flesh & 85.9%/grain)	sheep UK (87.0%/flesh & 85.8%/grain)
ms1483 #°2	sheep	calf DE (96.2%/flesh)	sheep UK (85.7%/flesh & 85.6%/grain)	sheep UK (85.8%/flesh & 85.5%/grain)
msmeh #°1	sheep or goat	goat UK (96.1%/grain)	sheep UK (81.4%/flesh & 81.7%/grain)	sheep UK (81.4%/flesh & 81.8%/grain)
msmeh #°2	sheep or goat	goat UK (96.1%/grain)	sheep UK (85.5%/flesh & 85.3%/grain)	sheep UK (85.6%/flesh & 85.5%/grain)
msfkat #°1	calf	calf DE (89.2%/grain)	calf DE (80.6%/flesh & 78.9%/grain)	calf DE (81.0%/flesh & 78.9%/grain)
msfkat #°2	calf	calf DE (89.2%/grain)	calf DE (80.5%/flesh & 77.3%/grain)	calf DE (80.6%/flesh & 77.2%/grain)
ms1240 #°1	sheep or calf	calf UK (95.3%/grain)	calf DE (81.6%/flesh & 80.7%/grain)	calf DE (81.8%/flesh & 80.6%/grain)
ms1240 #°2	sheep or calf	calf UK (95.3%/grain)	calf DE (85.3%/flesh & 84.4%/grain)	calf DE (86.0%/flesh & 84.5%/grain)
ms1556 #°1	sheep	calf DE (98.2%/flesh)	sheep UK (87.2%/flesh & 85.9%/grain)	sheep UK (87.3%/flesh & 86.0%/grain)
ms1556 #°2	sheep	calf DE (98.2%/flesh)	sheep UK (86.9%/flesh & 85.9%/grain)	sheep UK (87.0%/flesh & 86.0%/grain)
ms1482 #°1	sheep	sheep UK (94.0%/grain)	sheep UK (87.1%/flesh & 84.8%/grain)	sheep UK (87.2%/flesh & 84.9%/grain)
ms1482 #°2	sheep	sheep UK (94.0%/grain)	sheep UK (86.2%/flesh & 83.3%/grain)	sheep UK (86.3%/flesh & 83.3%/grain)
ms499 #°1	sheep	calf UK (96.5%/grain)	sheep UK (81.8%/flesh & 82.0%/grain)	sheep UK (81.9%/flesh & 81.9%/grain)
ms499 #°2	sheep	calf UK (96.5%/grain)	sheep UK (83.7%/flesh & 83.5%/grain)	sheep UK (83.8%/flesh & 83.5%/grain)
msopph #°1	sheep	sheep UK (96.5%/grain)	sheep UK (86.7%/flesh & 84.0%/grain)	sheep UK (86.8%/flesh & 84.1%/grain)
msopph #°2	sheep	sheep UK (96.5%/grain)	sheep UK (86.6%/flesh & 85.2%/grain)	sheep UK (86.7%/flesh & 85.2%/grain)

*Ms is the abbreviation for manuscript.

**See explanations in text.

In order to assess this new recognition method, proteomics tests were performed on all historical samples (Table 2, 2^nd^ column). The method was found to match up to 50% of the species. The reason for discrepancies is the fact that only PC1 and PC2 scores were used for recognition: a choice that turned out to be too restrictive to catch the full information contained in optical measurements. The recognition method failed in those cases where the PC1 percentage variability explained (PVE) was lower than 96.7%, as determined by examination of all our results.

Trying to solve the unmatched cases, we discovered that perfect matching, i.e. species identification and not simply species recognition, could be achieved using full PC scores (matrix size: (*M* − 1), see details in Methods), since they only fully account for the complex interaction of light with parchment when considered together. Contrary to the previous recognition approach, separate *X* matrices were built for the historical parchment under test and all modern parchments, considering grain side and flesh side separately. The historical sample matrix consisted of *N* = 2150 variables and *M* = 5 × 2 = 10 observations (*p* = 5 spectral measurements and *n* = 2 samples measured on only one side). Each modern parchment matrix (for either grain or flesh side) had size that varied according the number of samples available for each species. Identification works as follows. Given ${{\rm{Y}}}_{known}^{{\rm{S}}}$ and Y_*unknown*_, the full PC scores of modern and historical observations respectively, the Euclidian distance between the historical parchment under test and the four available modern parchment sources (either flesh side or grain side) is calculated, i.e. ${d}_{s}=\Vert {{\rm{Y}}}_{known}^{{\rm{S}}}-{{\rm{Y}}}_{unknown}\Vert$. The smallest distance is regarded as the criterion for species identification. Moreover, a proximity percentage is defined for each calculated distance, $\% {\rm{P}}=100-({d}_{s}\times 100/{\sum }_{m=1}^{4}{d}_{m})$, where ${\sum }_{m=1}^{4}{d}_{m}$ is the sum of historical parchment distances to the four modern parchment sources (S = {sheep UK, calf UK, goat UK, calf DE}). The proximity percentage ranges from 100% to 60.7%. The 100% value is the trivial case of the self-distance of a parchment and the 60.7% threshold value is the smallest value among distances between flesh side and grain side of modern parchments. Animal species identification is considered as successful only if the calculated proximity value is above that threshold. The number of samples for each modern parchment was greater than the number of samples available for each historical parchment. However, the former had to be the same as the latter in order to calculate the norm. We therefore selected randomly sets of ${[A,T,{T}_{d},R,{R}_{d}]}_{\alpha }$ within ${{\rm{Y}}}_{known}^{{\rm{S}}}$, in order to match the size of Y_*unknown*_, so that the same number of PCs was obtained in both cases. Remarkably, the method provided 100% of correspondence with proteomics results (Table 2, 4^th^ column).

Furthermore, in order to test the robustness of the proposed method, instead of doing the PCA using all samples for modern each parchment source, as above, we reduced the number of modern samples (random choice of 2 among several ones) to match that of the historical parchment. Thereafter, we recalculated ${{\rm{Y}}}_{known}^{{\rm{S}}}$ (*M* = 10 and *N* = 2150). The results were found to be almost identical to those obtained with the previous procedure (Table 2, 5^th^ column): an outstanding outcome if we consider that random selection affects PC scores differently in both procedures. This proves the robustness of the proposed method. As a matter of fact, using all PCs instead of only the first two ensures that all principal characteristics of the interaction of light with parchment were taken into account, allowing accurate species identification in 20 historical parchments, dating from 12^th^ century to 16^th^ century.

## Discussion

Herein, we present, for the first time, a dimensionality reduction method applied to a set of observations made on parchments, involving spectral measurements of optical properties such as transmittance, reflectance and absorbance. Those measurements are non-invasive and provide relevant information in the frequency (wavelength) domain. Spectral information is organized and processed by principal component analysis, leading to an innovative method for animal origin species identification in parchments. Optical radiation scattered by the sample is captured by an integrating sphere. The transmittance, reflectance and absorbance spectra recorded from a sample are a fingerprint of both the animal skin and the parchment manufacture. However, direct comparison of spectra from known parchment sources with those of historical parchments is a difficult task, since aging modifies material properties according to the parchments’ history, leading to spectral modifications. Thus, retrieving the principal characteristics of parchments from their optical properties is a fundamental step towards achieving species recognition and identification. Data processing is primarily based on the analysis of spectral variations. For this purpose, spectra are organised in a data matrix *X* and the corresponding correlation matrix *S*_*X*_ is transformed into an uncorrelated data set via the principal components matrix *P*, yielding a score matrix *Y* = *PX*.

Overall, the results depend on the observations inserted into the *X* matrix. For instance, species recognition is achieved by calculating the norm between centroid vectors of clusters, the components of which are the two first PCs of the *X* matrix built from absorbance, total reflectance and diffuse reflectance measurements of all modern parchments and of the historical parchment under test. This procedure does not produce full success in species recognition since it involves only the variances of the most relevant principal characteristics of the interaction of light with parchment. Species identification, on the other hand, is performed based on the shortest Euclidean distance between *all* PC scores from a specific element of the species database with those of the historical parchment under test. The specific interaction of light with parchment enables species identification through the proposed method. Animal species does not solely determine the optical properties of a parchment: manufacture matters greatly also. Indeed, calf parchment supplied by Cowley (UK) is distinguishable from calf parchment supplied by Schmedt (DE), either by visual inspection and optical measurements or through PCA representation (Fig. 3). Actually, our method enables species identification with additional discrimination based on parchment manufacture method. By contrast, proteomics and genetics work at the molecular level, hence are unable to make difference between two manufacturing processes applied to the same animal skin.

In conclusion, the results obtained by our method are reliable. That reliability comes from energy conservation in light scattering phenomena and, in particular, from distinctive features brought by animal origin species in those phenomena. The method described above compares favourably with genetics and proteomics, providing a unique optical fingerprint of the parchment’s species of origin. It is non-invasive, straightforward to implement, potentially cheap and accessible to scholars and conservators, with minimal training. Finally, it could help solving questions related to parchment production and, more generally, medieval writing production.

## Methods

### Parchment samples

Both modern and historical parchments were provided by the Moretus Plantin University Library (BUMP) of the University of Namur, Belgium. Modern parchments came from two suppliers: Cowley Co., United Kingdom (hereafter named Cowley, UK) and Schmedt GmbH & Co., Germany (hereafter named Schmedt, DE). They were made from three animal species: *Capra hircus* (goat), *Ovis aries* (sheep) and *Bos taurus* (calf). There were 21 samples in total: 6 goat parchment samples, 6 calf parchment samples and 5 sheep parchment samples from Cowley and 4 calf parchment samples from Schmedt. Ten historical parchments were also provided by BUMP for the present study. We were allowed to take 2 samples from each parchment (20 samples in total). The oldest parchment dated from twelfth century (ms499) and the most recent one from sixteenth century (ms1593).

### Optical measurements

A commercial double-beam spectrophotometer (Perkin Elmer, LAMBDA 750) equipped with an integrating sphere was used for optical measurements. The role of the sphere was to spatially integrate radiant flux coming from the parchment sample. The largest available sphere diameter (150 mm) was used because it provided better light integration and was less affected by measurement errors due to radiant flux losses. Diffuse standard coating covered the inner wall of the sphere as well as the elements used to block the ports. Measurements were performed across a wide spectral range (200 nm to 2350 nm) covering UV, visible and near infrared regions, at speed scan of 283.50 nm/min. Parchment samples were measured at a single position on both sides, indistinguishable for historical parchments but identifiable as flesh and grain sides for modern parchments. Total transmittance or reflectance measurements included both specular and diffuse components of the radiation scattered by the sample, whereas diffuse transmittance or reflectance measurements excluded the specular component. The sphere had 3 ports: entrance port (P1), exit port (P2) and off-axis port (P3). For measuring total transmittance (*T*), the sample was placed at the front of P1 and P2 and P3 were closed (Fig. 1b). For measuring diffuse transmittance (*T*_*d*_), P2 was open so that the specular component was trapped on exiting the sphere. For measuring total reflectance (*R*), the sample was placed at the rear of the sphere (P2) and P3 was closed (Fig. 1b). For measuring diffuse reflectance (*R*_*d*_), P3 was open so that the specular component exited the sphere at a tilt angle of 8 degree. All these measurements were non-invasive as they only required placing the sample in contact with either P1 or P2. For measuring absorbance (*A*), the sample was placed on a holder inside the sphere, with P2 and P3 closed. Absorbance is related to sample absorption (*a*) through: $A=-\,lo{g}_{10}(1-a)$. For that measurement, small pieces (size: 7.5 cm×5.0 cm for modern parchments and 3 cm×2 cm for historical parchments) had to be cut from parchments in order to fit within the sphere. However, absorbance measurements were not strictly necessary since absorption could always be deduced from *T* and *R* measurements, by virtue of the energy conservation law: *a* = 1-(*T* + *R*). In the present study, they were performed for comparison purpose only. In total, 210 (200) measurements were done for modern (historical) parchments, respectively.

### Principal Component Analysis (PCA)

Spectral measurements recorded from parchments are organized in a data matrix *X* of size *M* × *N* (*M*: number of measurements, *N*: number of wavelengths). The number of measurements has to be defined by the user, according to the targeted analyses. *X* matrix elements are organized by groups using submatrices noted *X*_*α*_ (size *p* × *N*) where *α* identifies any parchment sample, indexed as *α* = 1, …, *n* (*n*: total number of samples).

1 $${{\rm{X}}}_{{\rm{\alpha }}}=(\begin{array}{ccc}{{\rm{q}}}_{1}^{{\rm{\alpha }}}({{\rm{\lambda }}}_{1}) & \cdots  & {{\rm{q}}}_{1}^{{\rm{\alpha }}}({{\rm{\lambda }}}_{{\rm{N}}})\\ \vdots  & \ddots  & \vdots \\ {{\rm{q}}}_{{\rm{p}}}^{{\rm{\alpha }}}({{\rm{\lambda }}}_{1}) & \cdots  & {{\rm{q}}}_{{\rm{p}}}^{{\rm{\alpha }}}({{\rm{\lambda }}}_{{\rm{N}}})\end{array})$$

Here, ${{\rm{q}}}_{{\rm{p}}}^{{\rm{\alpha }}}$ represents any of the five available types of measurements (1 ≤ *p* ≤ 5), namely *A*, *T*, *T*_*d*_, *R*, *R*_*d*_. The number of rows of *X* is therefore given by *n* × *p* = *M*. PCA works on the correlation matrix of correlated variables of those observations, ${S}_{X}=\frac{1}{N-1}X{X}^{t}$ (*t* denotes matrix transposition), in order to produce uncorrelated variables, i.e. to eliminate data redundancy. For that purpose, a linear transformation *P* is applied on the original data matrix, i.e. *PX* = *Y*. This leads to a score matrix *Y*, which is a new representation of the original data using *P* as an orthonormal basis set formed by the principal components. Each principal component (PC) is associated with a variance *σ*^2^ which is the corresponding diagonal element of the correlation matrix ${S}_{Y}=\frac{1}{N-1}Y{Y}^{t}$.

### Software used for PCA

The program used for computing PCA is a code (pca.m) developed by MATLAB®. By default, the PCA code centers raw data of the input matrix *X* and computes the principal component coefficients matrix *P* (“coeff”), known as *loadings*, using singular value decomposition (SVD). The rows of *X* are the observations (here optical measurements) and the columns correspond to the variables (here wavelengths). The PCA code also returns a new representation of the input matrix in the principal components space, i.e. the principal component scores matrix *Y* (“score”) as well as the principal component variances (“latent”). The later are the eigenvalues of the covariance matrix *S*_*X*_, to each of which are associated a percentage (“explained”). Principal components are ordered by decreasing variances. Each row of the score matrix is plotted in the two-dimensional PC1-PC2 space as a point using bicaplot.m code, a modified version of the original biplot.m code by MATLAB®. The “bicaplot” code was developed for a better visualization of the data.

### Proteomic analyses

Samples were prepared following the ZooMS method. The parchment surface was gently rubbed with an eraser and eraser crumbs containing collagen molecules detached from the surface were place into tubes. Collagen was then extracted in solution and digested with trypsin. After those preparation steps, samples were analysed using liquid chromatography (UltiMate 3000 (ThermoFisher) coupled to electrospray tandem mass spectrometry (maXis Impact UHR-TOF (Bruker)) (LC-MSMS). Peptides from protein digestion were separated by reverse-phase liquid chromatography using a 75 µm × 150 mm column (Acclaim PepMap 100 C18). Mobile phase A was composed of 95% H_2_O, 5% ACN, 0.1% formic acid. Mobile phase B was composed of 80% CAN, 20% H_2_O, 0.1% formic acid. After injection of the peptides digest, the gradient started linearly from 5% B to 40% B in 15 min and from 40% B to 100% B in 5 min. The column was directly connected to a Captive Spray source (Bruker). In survey scans, mass spectrometry (MS) spectra were acquired for 0.5 s in the m/z range between 50 and 2200. The most intense peptides (2^+^ or 3^+^ ions) were sequenced during a cycle time of 3 seconds. The collision-induced dissociation (CID) energy was automatically set according to mass to charge (m/z) ratio and charge state of the precursor ion. MaXis and Ultimate systems were piloted by Compass HyStar 3.2 (Bruker). Peak lists for all samples were created using DataAnalysis 4.0 (Bruker) and saved as mgf file for use with ProteinScape 3.1 (Bruker). Mascot 2.4 (Matrix Science) was used as the search engine for the protein identification. Enzyme specificity was set to trypsin, and the maximum number of missed cleavages per peptide was set to one. Hydroxylation (KP) and oxidation (M) were allowed as variable modification. Mass tolerance for monoisotopic peptide window was 7 ppm and MS/MS tolerance window was set to 0.05 Da. For the protein identification, Mascot used a home-made collagen protein database and a contamination protein database. Scaffold software (Proteome Software) was used to validate protein and peptide identifications and also to perform the search of species marker peptides. Home-built species marker database contained specific peptides that allowed us to differentiate between *Capra hircus* (goat), *Ovis aries* (sheep) and *Bos Taurus* (calf).

## Supplementary information

Animal species identification in parchments by light, Angel Martin Fernandez Alvarez, Julie Bouhy, Marc Dieu, Catherine Charles and Olivier Deparis (Supplementary information)
