# Supplementary material for: Animal species identification in parchments by light
Source: Sci Rep. 2019 Feb 12;9:1825. doi: 10.1038/s41598-019-38492-z (PMC6372671; doi:10.1038/s41598-019-38492-z)
Supplement: Supplementary file 1 — Animal species identification in parchments by light, Angel Martin Fernandez Alvarez, Julie Bouhy, Marc Dieu, Catherine Charles and Olivier Deparis (Supplementary information) [file 41598_2019_38492_MOESM1_ESM.pdf]

Animal species identification in parchments by light

Angel Martin Fernandez Alvarez, Julie Bouhy, Marc Dieu, Catherine Charles and Olivier Deparis

Supplementary Material

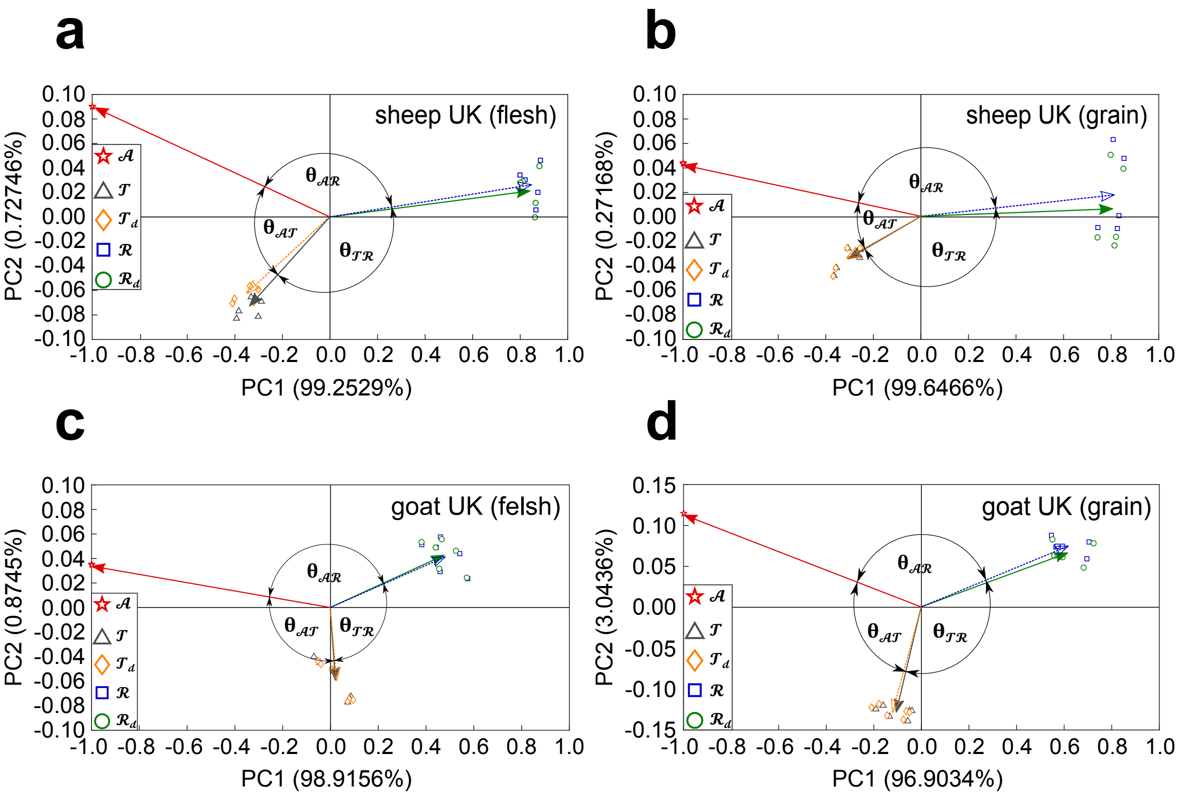

Fig. S1 | PCA data representation from A, T, T_d, R, R_d measurements on modern parchment samples. a-d, PCA representation of modern (Cowley, UK) parchment data: sheep (**a,b**) and goat (**c,d**). Flesh side (**a,c**) and grain side (**b,d**). A centroid vector is calculated for each type of measured quantity and the angle θ between centroid vectors is related to Pearson coefficient by $r = \cos\theta$. For each PCA representation, the percentage variability explained (PVE) is displayed on each axis.

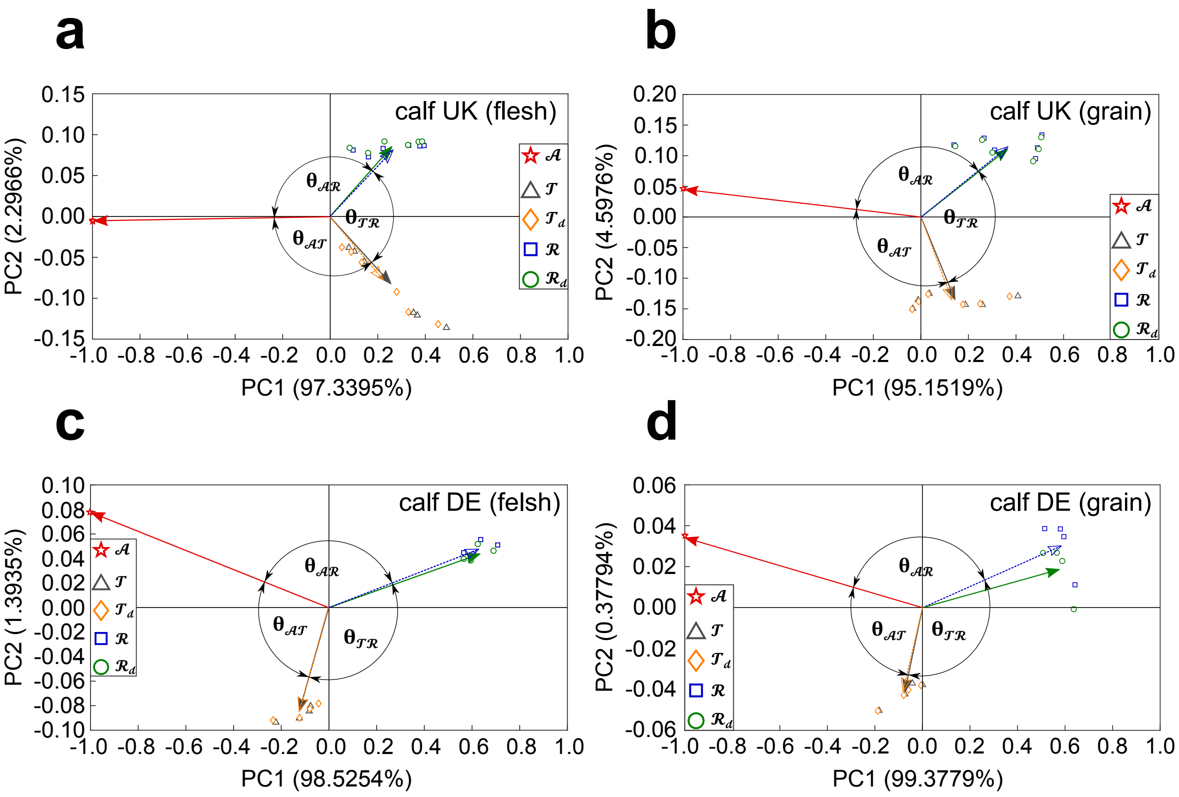

Fig. S2 | PCA data representation from A, T, T_d, R, R_d measurements on modern parchment samples. a-d, PCA representation of modern parchment data: calf form Cowley, UK (**a,b**) and calf from Schmedt, DE (**c,d**). Flesh side (**a,c**) and grain side (**b,d**). A centroid vector is calculated for each type of measured quantity and the angle θ between centroid vectors is related to Pearson coefficient by $r = \cos\theta$. For each PCA representation, the percentage variability explained (PVE) is displayed on each axis.

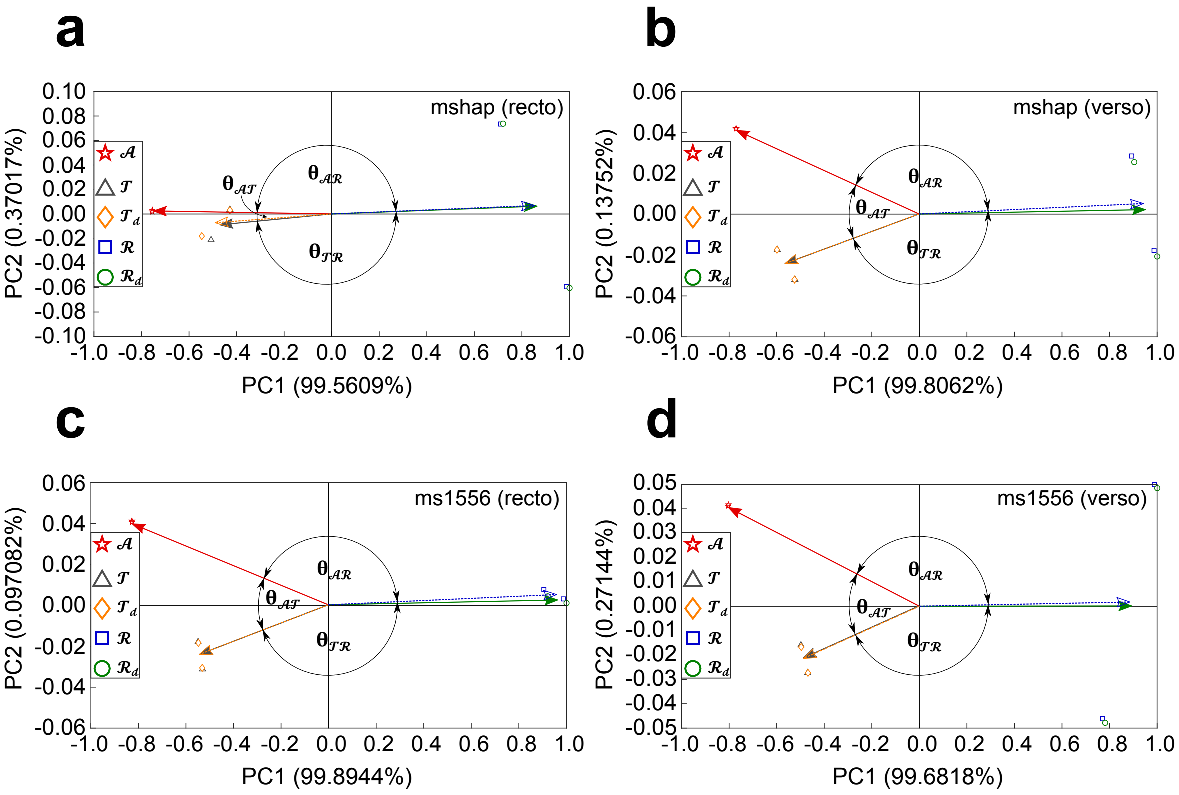

Fig. S3 | PCA data representation from A, T, T_d, R, R_d measurements on historical parchment samples. a-d, PCA representation of historical parchment data: mshap (**a,b**) and ms1556 (**c,d**). Recto (**a,c**) and verso (**b,d**). A centroid vector is calculated for each type of measured quantity and the angle θ between centroid vectors is related to Pearson coefficient by $r = \cos\theta$. For each PCA representation, the percentage variability explained (PVE) is displayed on each axis.

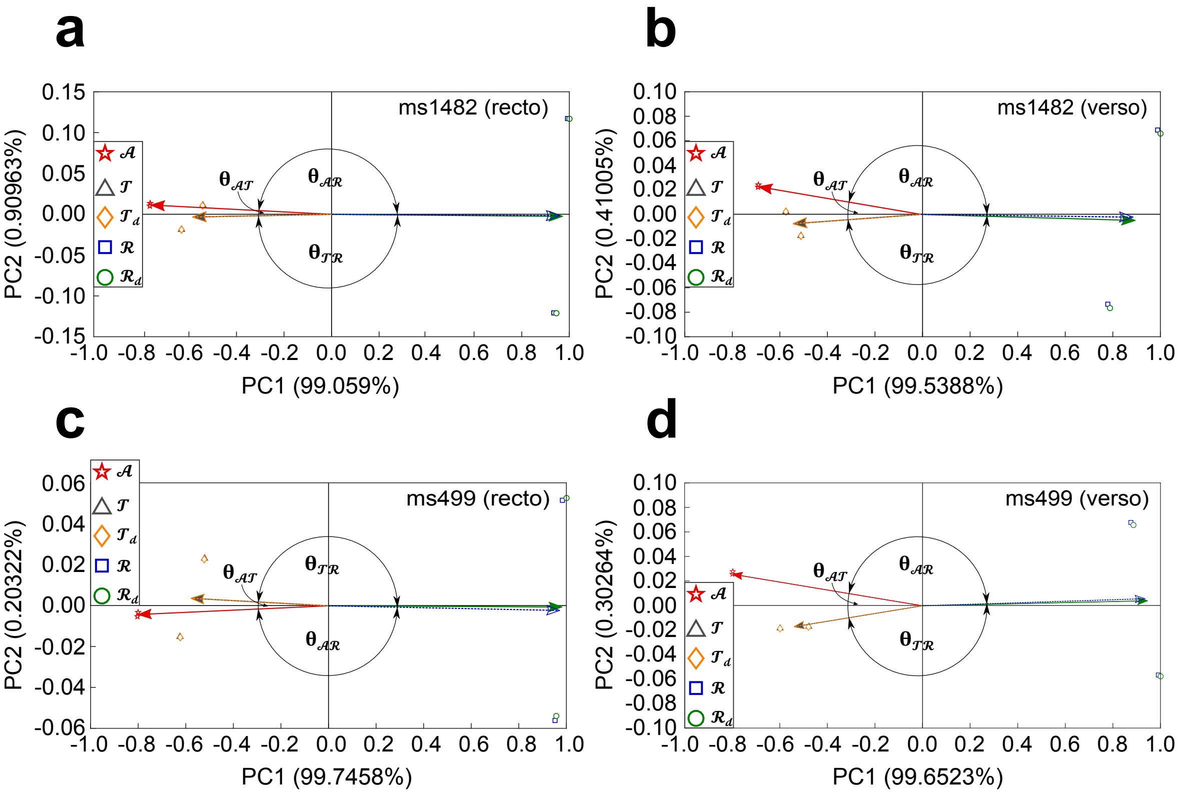

Fig. S4 | PCA data representation from $\boldsymbol{A}$, $\boldsymbol{T}$, $\boldsymbol{T_d}$, $\boldsymbol{R}$, $\boldsymbol{R_d}$ measurements on historical parchment samples. a-d, PCA representation of historical parchment data: ms1482 (**a,b**) and ms499 (**c,d**). Recto (**a,c**) and verso (**b,d**). A centroid vector is calculated for each type of measured quantity and the angle θ between centroid vectors is related to Pearson coefficient by $r = \cos\theta$. For each PCA representation, the percentage variability explained (PVE) is displayed on each axis.

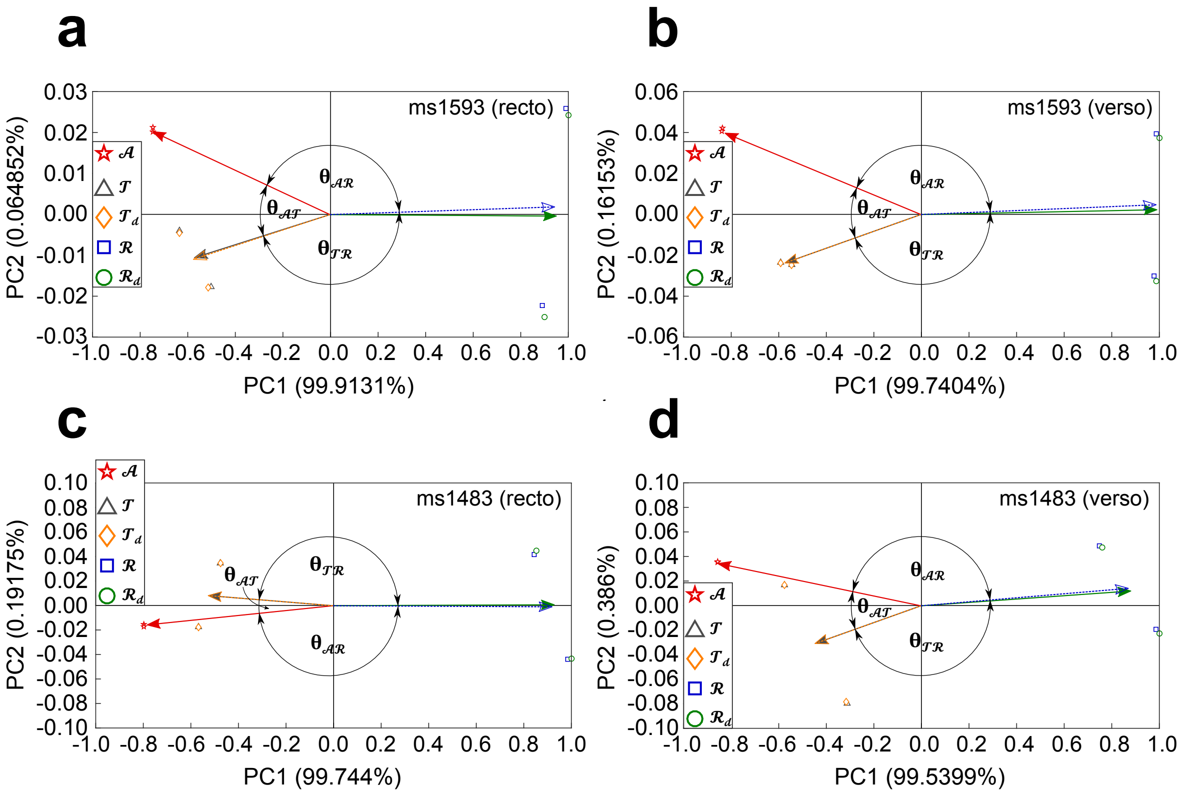

Fig. S5 | PCA data representation from $\boldsymbol{A}$, $\boldsymbol{T}$, $\boldsymbol{T}_d$, $\boldsymbol{R}$, $\boldsymbol{R}_d$ measurements on historical parchment samples. a-d, PCA representation of historical parchment data: ms1593 (**a**,**b**) and ms1483 (**c**,**d**). Recto (**a**,**c**) and verso (**b**,**d**). A centroid vector is calculated for each type of measured quantity and the angle θ between centroid vectors is related to Pearson coefficient by $r = \cos\theta$. For each PCA representation, the percentage variability explained (PVE) is displayed on each axis.

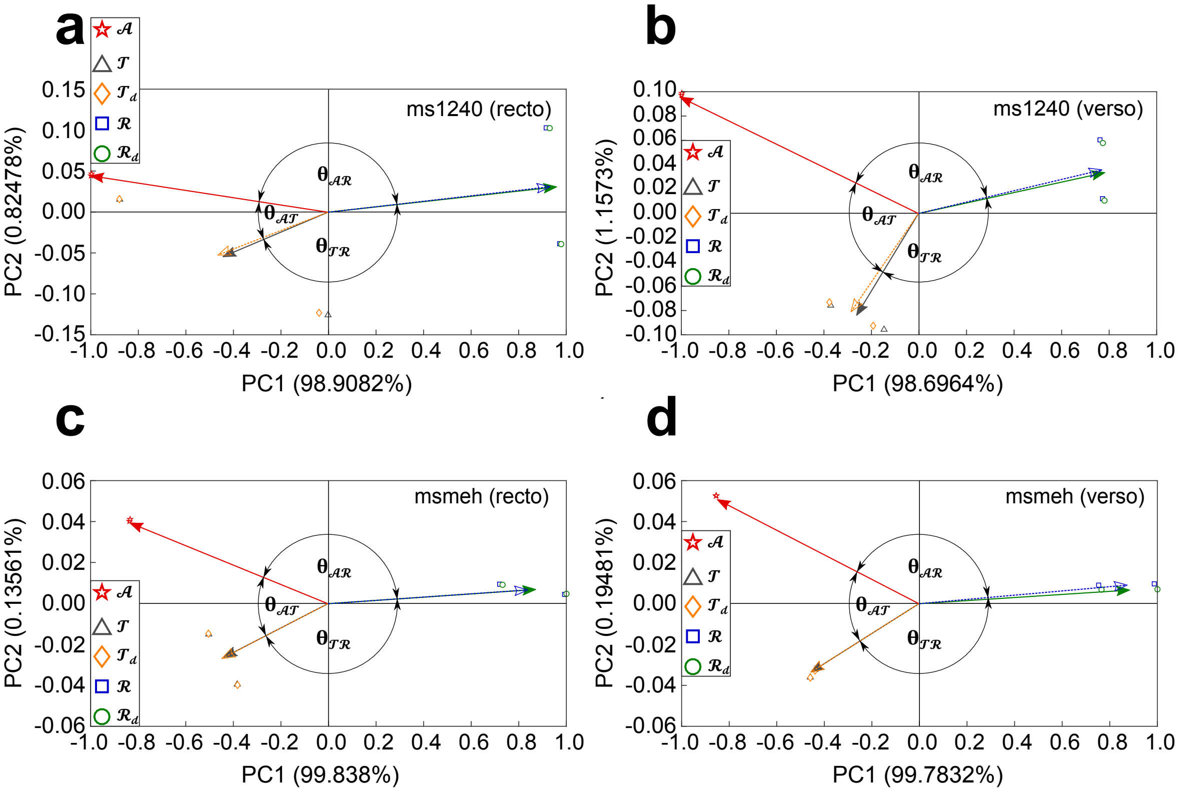

Fig. S6 | PCA data representation from A, T, T_d, R, R_d measurements on historical parchment samples. a-d, PCA representation of historical parchment data: ms1240 (**a,b**) and msmeh (**c,d**). Recto (**a,c**) and verso (**b,d**). A centroid vector is calculated for each type of measured quantity and the angle θ between centroid vectors is related to Pearson coefficient by $r = \cos\theta$. For each PCA representation, the percentage variability explained (PVE) is displayed on each axis.

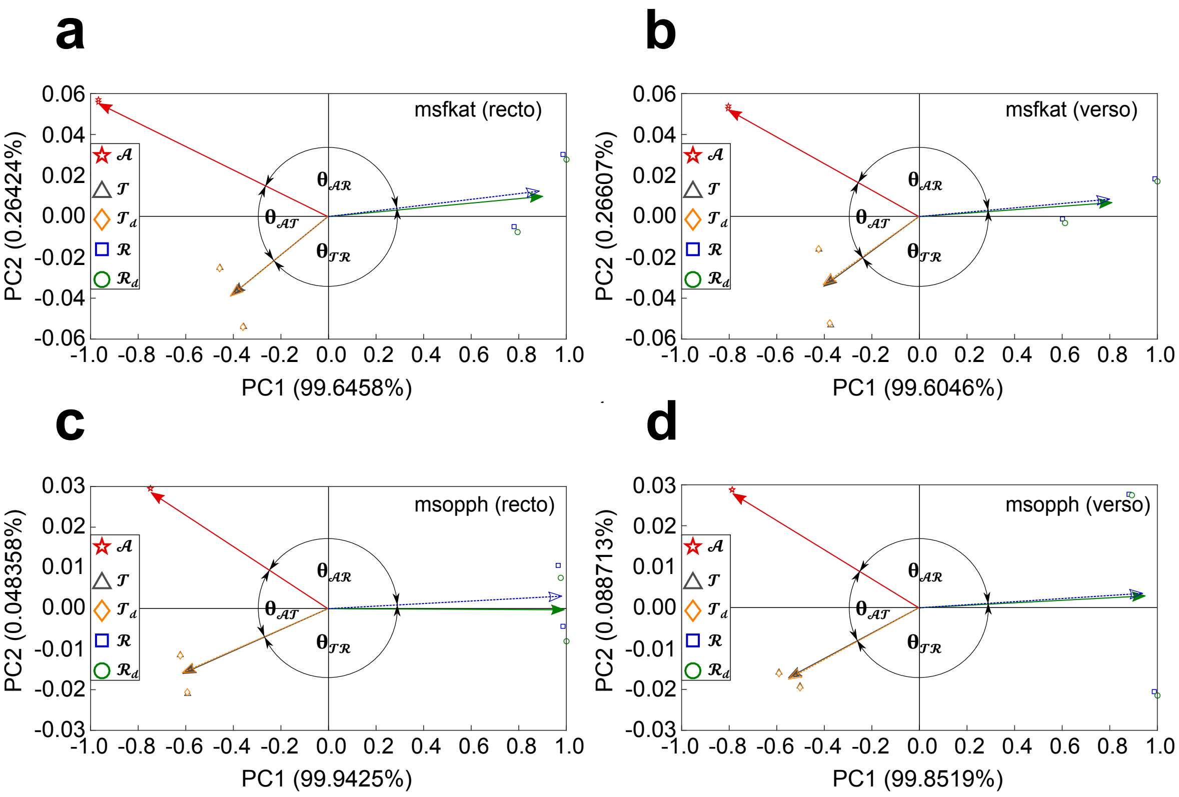

Fig. S7 | **PCA data representation from A, T, T_d, R, R_d measurements on historical parchment samples. a-d,** PCA representation of historical parchment data: msfkat (**a,b**) and msopph (**c,d**). Recto (**a,c**) and verso (**b,d**). A centroid vector is calculated for each type of measured quantity and the angle θ between centroid vectors is related to Pearson coefficient by $r = \cos\theta$. For each PCA representation, the percentage variability explained (PVE) is displayed on each axis.
